# Editorialets

**Published:** 1905-11

**Authors:** 


### Editorialets.

---

DR M. M. POOL, of El Campo, Texas, is doing post-graduate work in Chicago.

---

SURGEON D. L. PEEPLES, T. V. G., of Navasota, is doing post-graduate work in St. Louis.

---

THE EAST TEXAS MEDICO-CHIRURGICAL ASSOCIATION will meet in Palestine November 23 and 24. The meetings of this sterling organization are well attended and are always interesting. I will try to be there.

---

A CORRECTION. An article in "The Publisher's Department" in our September number on "Hemorrhage of the Bladder," in which Eusoma had been found effective, was, by a clerical error, credited to the *Medical World* instead of the *Medical Herald*.

Differential Diagnosis.—*American Medicine* says that in a recent text-book on the practice of medicine, there is a chapter headed, in big caps, "To distinguish chicken from smallpox."

---

Dr. McCormack at Austin. In pursuance of announcement, Dr. J. N. McCormack, of Bowling Green, Ky., Chairman of the A. M. A. Committee on Organization, addressed the Travis County Medical Society and numerous visiting physicians from adjoining counties on November 3d, inst. There were many others than physicians present, prominent public-spirited citizens, who listened with close attention and deep interest to the eloquent speaker's interesting and pleasing address. It was on County Medical Societies, the foundation and bulwarks of organized medicine; and the individual physician, his duties and obligations. The speaker was introduced by Dr. Daniel, ex-President of the State Medical Association, who presided over the meeting. In response to his invitation, short addresses were made by Col. A. P. Wooldridge, Judge J. H. B. Miller, Dr. Chase, Secretary State Medical Association; Dr. Bennett, Councilor Seventh District.

---

Dr. Joseph Price in Dallas. As per announcement in our last issue, Dr. Price, of Philadelphia, spent October 30 and 31 in Dallas, the guest of the Dallas County Medical Society. He was accompanied by Drs. Chas. E. Thomas and Thos. E. Parke, of Philadelphia. The party was treated with the most distinguished courtesy,—quite an ovation. Dr. Price delivered an extemporaneous address on Appendicitis.

At Dr. Leake's Sanitarium he operated for appendicitis in three cases; at the St. Paul Sanitarium he operated four times, once for renal calculus. At the Oriental Hotel a general reception was held and thousands of citizens other than doctors met the famous abdominal surgeon. He says that golf is a prolific source of appendicitis in women.

---

That Sure Cure for Consumption. The great Behring, the inventor and patentee of diphtheria antitoxin, at the recent Congress on Tuberculosis held at Paris, startled the world by an announcement—very premature—that he had discovered a sure cure for consumption; but until he can get it patented he is not going to "give it away"! It is said to be a white powder—he calls it "C. T." or "T. C.," and that it is made from the milk of tuberculous cows. However, that is mere conjecture. But it is interesting in connection with the startling announcement recently made on the highest authority that the "microbe" or "bacillus" of old age has been discovered and that sour milk will kill it and enable a person to live forever. The long-lived Bulgarians are cited as living proof of the claim and the statement is made that they live on sour milk and curds and whey. Moral: *Cheese it.* What will be the attitude of Dr. Simmons and other "unco gude" towards a patented consumption cure *a la* Behring and antitoxin?

---

FOR SALE.—Six-acre lot, good residence and outbuildings; situated in the town of Thornton, Texas. Terms easy. For further particulars, apply to T. M. Wilson, M. D., Thornton, Texas.

---

WHAT SOME OF THEM SAY.

"Here is my annual mite. The 'Red Back' is 'right up to now.' I'll 'be good' once a year. Keep up the good work and the ranks will do the rest."—NELSON, Richland Springs.

"I don't want to miss a single number of the 'Red Back.'"—TURNEY, Stoneburg, Texas.

"Success to the 'Red Back.'"—SPIVEY, Teneha.

"For the 'Red Back.' Best wishes. May you live forever to continue the good work of the ever welcome journal."—PANNELL, Martindale.

"I can not do without the 'Red Back.'"—TREADWAY, Miles.

"You are giving us some warm numbers. Just go ahead and keep it red hot."—SEALE, Marqueze.

"Here's to stimulate the 'Red Back' to renewed strength to shake off the Octopus. May the 'Red Back' thrive and grow redder and hotter with age."—WARREN, Snyder.

"Long may she flourish."—WHITE, Henderson.

"I read it carefully from cover to cover."—MARTIN, La Vernia.

"I take a number of journals, but like the 'Red Back' most of all."—MOORE, Caro, Texas.

PER CONTRA. Here's a wet hen. By a clerical error this subscriber was credited to May, 1905, instead of 1906. He writes:

"Chase, Texas, Oct. 2, 1905.

"Dr. F. E. Daniel, Austin.

Your notefication of my account received today. Now Dr. while I was at Houstin in April I paid for the Journal $1.00 when I Subscribed. Now if this is the Kind of Editor you are, You may

Send by return mail the balance (Subscribed from Dollar) Due me for the Price of Journal and Then Stop it right now. Now you do this and forever hold your Piece.

DR. J. R. PHILLIPS,
Chase, Texas.

